# On the difference in the rise times of the two SES electric field components

**Published:** 2004-06-01

**Authors:** Panayiotis A. Varotsos, Nicholas V. Sarlis, Efthimios S. Skordas

**Affiliations:** *) Solid State Section, Physics Department, University of Athens, Panepistimiopolis, Zofragos, 157 84, Athens, Greece; **) Solid Earth Physics Institute, Physics Department, University of Athens, Panepistimiopolis, Zofragos, 157 84, Athens, Greece

**Keywords:** Seismic Electric Signals, rise time, diffusion, electromagnetic fields

## Abstract

The study of low frequency signal transmission in conductive media, reveals that the electric and magnetic fields follow diffusion type equations. In a previous paper (Varotsos *et al*.1)) experimental evidence was forwarded that for epicentral distances of the order of 100 km, the SES electric field variations precede those of the magnetic ones by a time of the order of 1 sec. In the present paper, we present evidence that this peculiarity still pertains (but to a smaller extent), when studying the differences in the components of the electric field. This cannot be probably observed in the scale of laboratory measurements, lying usually within the error bars of the current experimental facilities. A tentative theoretical justification, termed as *τ*-approximation, is presented which accounts for the measurements of electric field components. The present findings can provide a unique tool for the discrimination between remote and nearby sources by using data from electric measurements alone.

## Introduction

In a previous paper, the analysis of the experimental data showed that the electric field variations associated with the SES precede those of the magnetic field recorded at DMM, by 1–2 sec (see Varotsos *et al*.1),2)). It is the object of this paper to show that the two electric field components, if properly measured, show measurable differences in their rise times. This difference was around 0.5 sec for the SES associated with the 6.6 Grevena-Kozani EQ recorded at IOA, i.e., at an epicentral distance r~80 km.

## Rise time

**Fig. 1:**
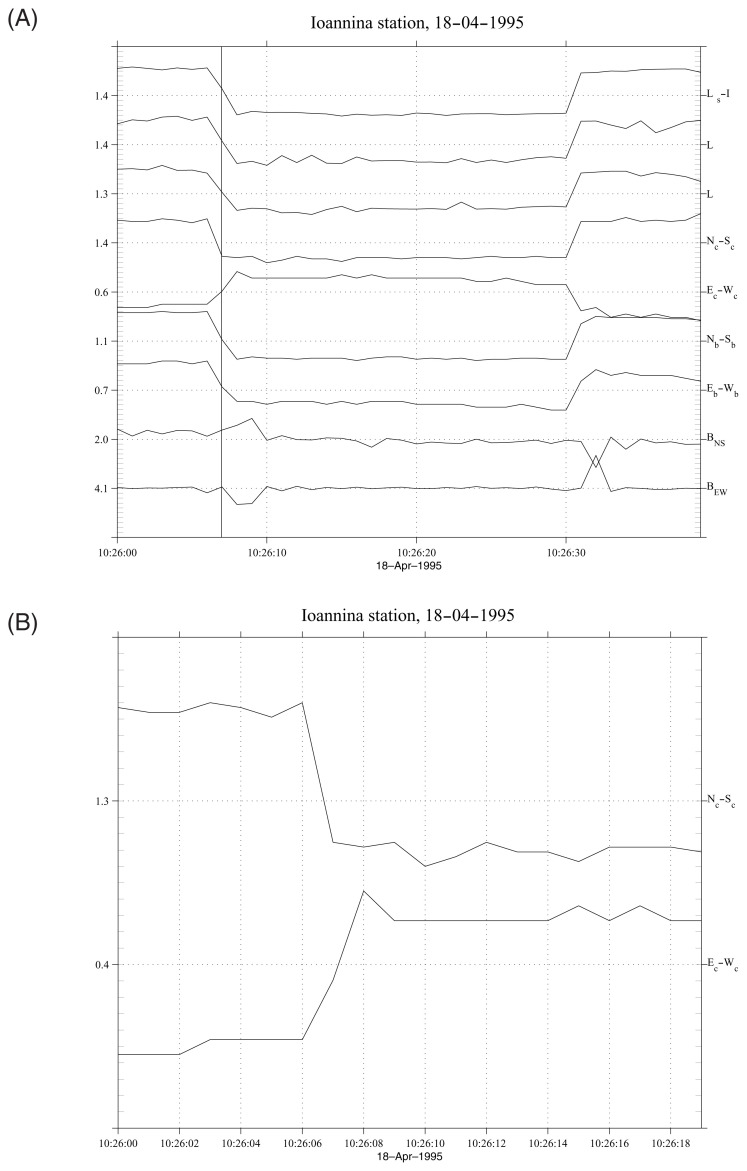
(A) Typical example of SES recordings at IOA on April 18, 1995. The vertical line is drawn just at the time when the “fast component” N_c_-S_c_ reaches its maximum (lowest in the figure) level; during this time E_c_-W_c_ still varies and maximizes in the next sampling (Note that these dipoles are oriented along and perpendicular to the direction of local channeling at site C, which does not occur for the dipoles N_b_-S_b_ and E_b_-W_b_). This is not observed for all the pulses but only for the percentages inferred from Table I. (B) The same as in A, but in a more expanded time scale, in order to show more clearly the difference in the rise times of N_c_-S_c_ and E_c_-W_c_.

The electric field measurements at the IOA station are made at F_s_ = 1 Hz sampling rate in a variety of long and short dipoles using Low Pass Filters (LPF) of two different kinds; those of 1 Hz and those of 10 Hz. Here we shall concentrate our interest on the short electric dipoles deployed in the areas B and C of the IOA station (see the map of Fig. 2 of Varotsos *et al*.3)). Beyond the dipoles deployed at both sites along the NS and EW direction, two dipoles labeled E’_b_-W’_b_ and N’_b_-S’_b_ are installed at site B along and perpendicular to the direction of the local channeling (Table I, see also Varotsos *et al*.4)). All these short dipoles were simultaneously measured by using the so-called 10 Hz LPF, when the SES activities of 18 and 19 April 1995 were recorded. Figure 1 shows an example of such recordings in an expanded time scale; in this example we can observe that the SES is composed of an almost abrupt (for F_s_ = 1 Hz) change in the electric field. However, there are pulses which exhibit a finite duration of their transition time from the background to the upper level and vice versa (i.e., sometimes they need 2 sec to reach the upper level from the background, or 2 sec from the upper level to reach the background). In order to study quantitatively this phenomenon, we define the SES rise time *τ*_r_ as the time needed for a SES pulse to reach the 85% of its maximum amplitude measured from the moment it has reached 15% (of its maximum amplitude). It is obvious that if we had measured SES with a higher sampling rate, we could directly estimate *τ*_r_ for each SES pulse. Since, however, such data are not available, we take advantage of the recording of a large number of SES pulses (approximately N = 500 pulses) to obtain an estimation for *τ*_r_. As a first approximation, the percentage of pulses N_f_/N with finite transition time (i.e. more than 1 sample) equals the ratio of *τ*_r_ over the sampling period (T_s_ = 1/F_s_). Table I shows the results obtained by such a procedure.

## Diffusion of electromagnetic fields in a heterogeneous medium

We consider, as a first approximation, the simplified case of a homogeneous conductive medium of conductivity *σ*_0_. A transient electric dipole I***l***, in this case, leads to the following unit step response of the current density field:

[1] $$j(r,\text{t})=[\text{s}(\text{t}/\tau_{0})(r\cdot \text{I}l)r-\text{f}(\text{t}/\tau_{0}){\text{r}}^{2}\;\text{I}l]/(4\pi {\text{r}}^{5})$$

where *τ*_0_ = *μ*_0_ *σ*_0_r^2^/4, while s(x) and f(x) stand for

[2] $$\text{s}(\text{x})=[4/{\text{x}}^{3/2}+6/{\text{x}}^{1/2}]\;\text{exp}[-1/\text{x}]/\sqrt{\pi }+3\;\text{erfc}[1/{\text{x}}^{1/2}],$$

[3] $$\text{f}(\text{x})=[4/{\text{x}}^{3/2}+2/{\text{x}}^{1/2}]\;\text{exp}[-1/\text{x}]/\sqrt{\pi }+\text{erfc}[1/{\text{x}}^{1/2}].$$

**Fig. 2:**
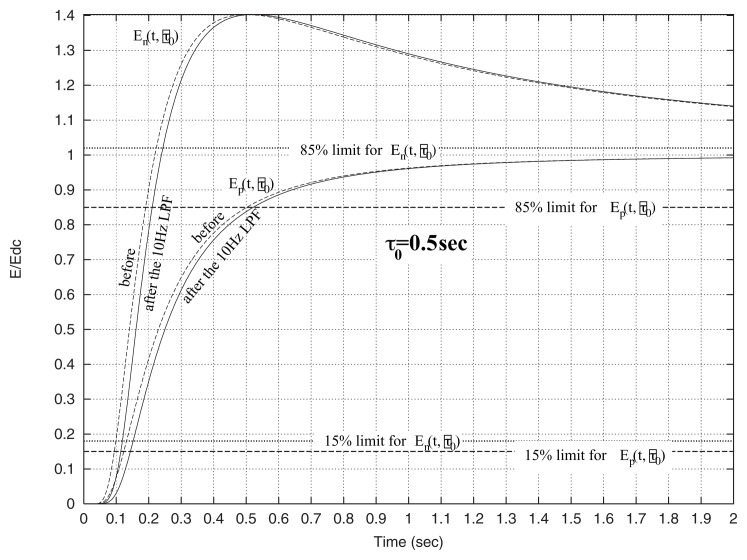
The time evolution of the fast, f(x), and slow, s(x), components (broken lines) of the electric field, which approximate E_n_ and E_p_ respectively (see the text later) for the case of *τ*_0_ = 0.5 sec. The solid lines correspond to values observed when using a 10 Hz LPF. The 15% and 85% are also drawn for each component.

These equations show that the two components of the electric field(one along **r** and one along the direction of the dipole I***l***) have time evolutions described by s(x) and f(x) respectively; they correspond to the slow and the fast evolution shown on Fig. 2. Thus, for a signal that has traveled a long distance through the solid earth crust, we expect, in principle, to find traces of this characteristic behavior.

The above holds, as mentioned, for a homogeneous conductive medium. In the case of the SES, however, the time evolutions of the electric field components must be calculated close to the edge of a highly conductive path (if the SES transmission model of Varotsos and Alexopoulos [1986]5) is envisaged). Such a calculation, however, is not currently available for the case when the current dipole is oriented almost perpendicular to the neighboring conductive path (which is likely to be the case for the SES emission, see Sarlis and Varotsos6)). We note that an analytical calculation (Varotsos *et al*. [2000]7)) for the case of a current dipole source lying inside and parallel to the main axis z of a conductive cylinder (radius R) of infinite length reveals the following: at long (reduced) distances from the emitting source, and for sites lying inside the more resistive medium but very close to the cylinder, the component E_n_ normal to its main axis has a rise time appreciably shorter than that of the parallel component E_p_. Their difference is of the order of ~1 sec for reasonable conductivity ratios, e.g. 4000/10, and for distances of the order of 100 km (R~500 m). It is intuitively expected that a similar behavior might also exist for the aforementioned more realistic case (i.e., for the components close to a conductive edge when the emitting dipole is oriented perpendicular to the conductive path). However, in view of the lack of an exact theoretical calculation for this heterogeneous structure, we now restrict ourselves to the comparison of the experimental data with the aforementioned time evolutions foreseen for the two components due to a dipole embedded in a homogeneous conductive medium. (This is not unreasonable, if we recall that at remote distances the field E_inside_ inside the conductive cylinder becomes equal to the field E_host_ in absence of the conductive cylinder.)

## Synthetic SES

**Fig. 3:**
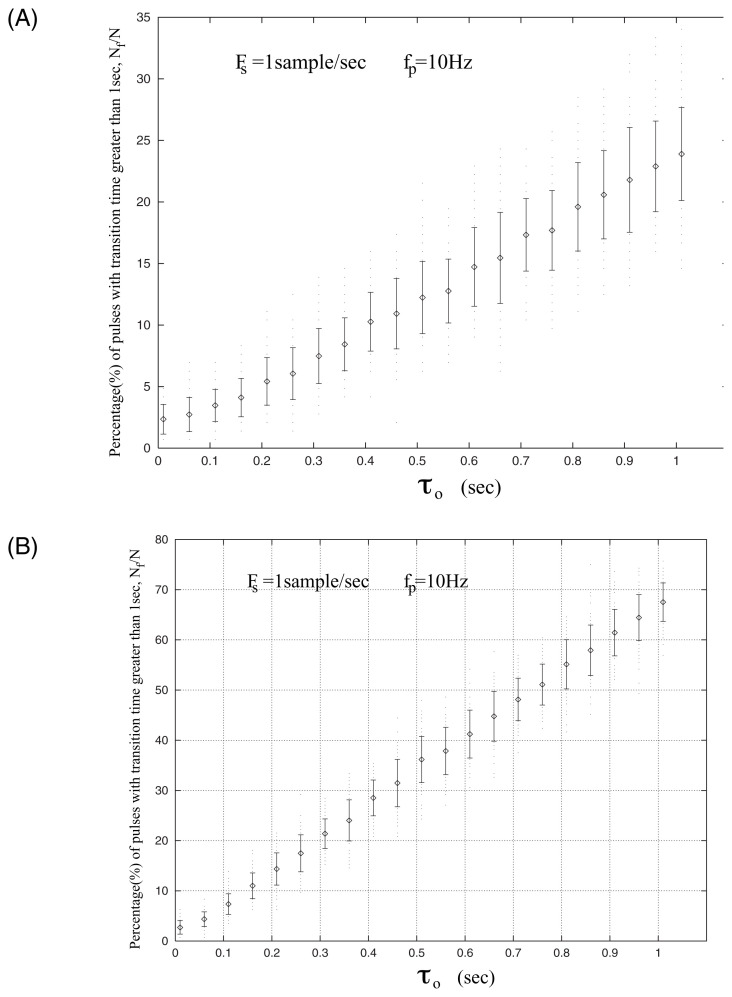
The percentage of pulses (N_f_/N) with “transition time” larger than 1 sec as a function of characteristic time *τ*_0_ as it results from the Monte Carlo simulation for sampling rate Fs = 1 Hz for a 10 Hz-low pass filter for A: E_n_ i.e., using f(x), and B: E_p_, i.e., using s(x).

In view of the recent laboratory results in the physics of molecular glasses (see Varotsos8),9)), it is highly probable that the SES emitted from the focal area have a form similar to Random Telegraph Signals (RTS). In order to produce synthetic SES, we consider a large number of boxcar pulses transmitted in the solid earth crust and simulated their recording at some distance from the source using the 10 Hz LPF. We assumed their edges are distorted according to either s(x) (i.e., E_p_) or f(x) (i.e, E_n_) given in Eqs. [2] and [3] for each value of *τ*_0_. We employed such a procedure for a series of 138 pulses emitted consecutively and randomly in time; this was repeated 100 times via Monte Carlo procedure and the results for the two components of the electric field are shown in Fig. 3.

## Discussion

Comparing the results included in Table I with those depicted in Fig. 3(a), (b), we find that the experimental data for the area C are compatible *for both components* of the electric field with the time evolutions estimated by Eqs. [2] and [3] for *τ*_0_~0.5 sec. Furthermore, we note that the data from area B, when rotated by N26 °W, also lead to the following estimations of N_f_/N:

$$\begin{array}{lll} \text{for} & \text{EW}(-26{}^{\circ}): & {\text{N}}_{\text{f}}/\text{N}=(36\pm 5)\%\\ \text{and for} & \text{NS}(-26{}^{\circ}): & {\text{N}}_{\text{f}}/\text{N}=(12\pm 3)\% \end{array}$$

These values are compatible, for both components of the electric field, with same value of *τ*_0_, i.e., *τ*_0_~0.5 sec.

The above results show that the recorded SES have “suffered” a diffusion corresponding to *μ*_0_*σ*_0_R^2^/4~0.5 sec where R denotes their source distance from IOA. This is compatible with R~80 km if the host resistivity is taken as being of the order of several thousand *Ω*m. Note that such a time of 0.5 sec is *not* compatible with any nearby source (e.g. with R < 10 km) even if we consider an average resistivity 100 *Ω*m (i.e., which is very small for the area of IOA). The same conclusions are drawn, if—instead of Eqs. [1]–[3]—we follow the analysis of ref. 10), which leads to *τ*_0_ ≈ 0.18 sec.

The above results indicate that one can obtain an estimation of the epicentral distance when making use of the independent measurements of the electric field components.

## Remark

**Fig. 4:**
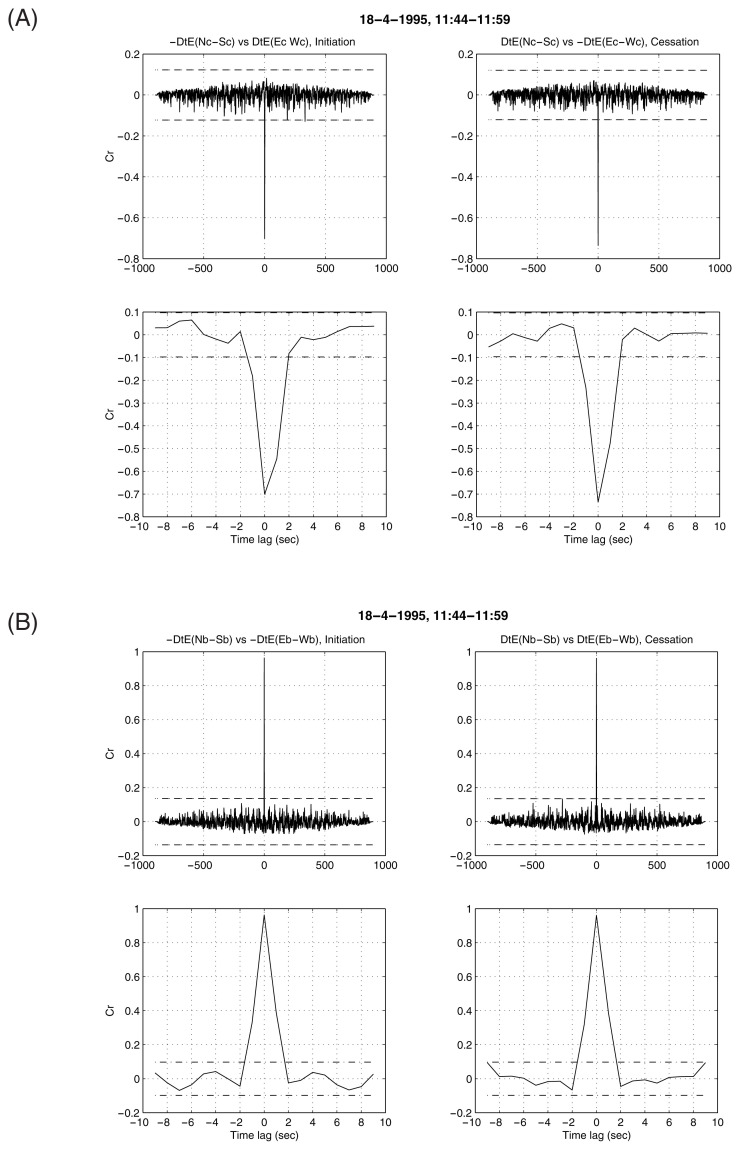
A: The calculated “cr” between the derivatives (Dt) of the two electric field components at site C versus their time-lag Δt. Upper row: values calculated without pre-whitening for Δt between −1000 sec and 1000 sec; second row: the “cr” values calculated as in the first row, but now depicted in an expanded time scale, i.e., between −10 and 10 sec; B: the same as in A but for the site B. Both figures refer to the main body of SES activity of April 18, 1995. Broken horizontal lines correspond to the 99% confidence level.

When comparing the two components at the site C, the following clarification is needed: What can be visualized in Fig. 1A, i.e., the N_c_-S_c_ reaches first the maximum deflection while E_c_-W_c_ still varies (and reaches its maximum at the next sampling), happens only in the percentage of cases inferred from Table I. In other words, in most cases these two electric components appear to have almost simultaneous initiation and cessation. This can be verified when calculating the correlation coefficient “cr” between N_c_-S_c_ and E_c_-W_c_: a prominent peak appears at a time-lag Δt equal to 0, but a secondary statistically significant correlation still pertains at Δt = 1 sec (see Fig. 4A). Such an “asymmetry” around Δt = 0 can also be recognized when calculating the “cr” between N_c_-S_c_ and N_b_-S_b_ (see Fig. 3 of Varotsos *et al*.7)). We emphasize the following distinction. At site B, as mentioned, none of the N_b_-S_b_ and E_b_-W_b_ dipoles have been installed to be exactly perpendicular to the local channeling and hence both components, at first glance, seem to be more or less equally “slow” (see Table I); thus, their corresponding “cr” does not exhibit any obvious asymmetry around Δt = 0 (Fig. 4B), in contrast to that observed for the case at site C.

## Conclusions: Outlook for the future

The content of the present paper, in conjunction to the other recent advances on our understanding on the physics of SES, may indicate that the future improvements of the capability of SES to predict earthquakes could include the following:

### Improving the epicenter determination

Beyond the gradual completion of the selectivity maps and the improvement of the calibration of each station for each seismic area, the following two possibilities could be envisaged: (1) the simultaneous measurement of the SES electric field variations and their accompanying magnetic field ones can lead to the determination of their time lag, therefrom allowing an estimation of the epicentral distance d (as described by Varotsos *et al*.7)) (2) in case that only measurements of the electric field are available, the study of the SES rise time, as well as its variation versus the measuring direction, may lead to an estimation of the distance d. Note that, once a good estimation of d is possible, this would allow a better estimation of the expected magnitude.

### Improving the accuracy of the time lag of the expected earthquake

As described in the accompanying paper11) (see also Varotsos *et al*.12),13)) this could be achieved as follows: Once a SES activity has been recorded, we proceed to its Fourier analysis, but in the “natural time domain”,12) and hence construct its power spectrum. Then, upon following the time evolution of the subsequent seismicity, we may identify the period when the power spectrum of the latter collapses (coming from below) on the spectrum of the former. This happens just a few days before the main shock.

## Figures and Tables

**Table I:** The percentage of SES pulses that have a finite transition time N_f_/N together with the corresponding estimation of *τ*_r_ for each of the dipoles presented in text. (The errors have been drawn using the error estimation by the Monte Carlo method described in text).

Dipole	N_f_/N (%)	*τ*_r_(msec)
L’_s_-I	15 ± 3	150 ± 30
L’	23 ± 4	230 ± 40
L	19 ± 4	190 ± 40
N_c_-S_c_	10 ± 2	100 ± 20
E_c_-W_c_	35 ± 5	350 ± 50
* N_b_’-S_b_’	17 ± 3	170 ± 30
* E_b_’-W_b_’	43 ± 5	430 ± 50
N_b_-S_b_	26 ± 4	260 ± 40
E_b_-W_b_	41 ± 5	410 ± 50

* Repeated long time MT measurements at site B at IOA showed that there is channeling along the direction N22 °W (to N26 °W). The dipole labeled N_b_’-S_b_’ and E_b_’-W_b_’ are installed at site B of IOA parallel with and perpendicular to this direction. Thus they are oriented along NS(−22 °) and EW(−22 °) respectively, i.e., after rotating the original axes by 22 ° anticlockwise.

## References

[1] Varotsos P. Sarlis N. Skordas E. (2001) Proc. Jpn. Acad., Ser. B 77, 93–97. 10.2183/pjab/84.331 PMC3722020 18941306

[2] Varotsos P. Sarlis N. Skordas E. (2001) Proc. Jpn. Acad., Ser. B 77, 87–92. 10.2183/pjab/84.331 PMC3722020 18941306

[3] Varotsos P. Lazaridou M. Eftaxias K. Antonopoulos G. Makris J. Kopanas J. (1996) In The Critical Review of VAN: Earthquake Prediction from Seismic Electric Signals (ed. Lighthill J.). World Scientific, Singapore, pp. 29–76.

[4] Varotsos P. Eftaxias K. Lazaridou M. Antonopoulos G. Makris J. Poliyiannakis J. (1996) Geophys. Res. Lett. 23, 1449–1452.

[5] Varotsos P. Alexopoulos K. (1986) Thermodynamics of point defects and their relation to bulk properties. North Holland, Amsterdam.

[6] Sarlis N. Varotsos P. (2001) Acta Geophys. Pol. 49, 277–285.

[7] Varotsos P. Sarlis N. Skordas E. (2000) Acta Geophys. Pol. 48, 263–297.

[8] Varotsos P. (2004) The Physics of Seismic Electric Signals (to be published).

[9] Varotsos P. (2001) Acta Geophys. Pol. 49, 1–42.

[10] Varotsos P. Sarlis N. Lazaridou M. (1999) Phys. Rev. B 59, 24–27.

[11] Tanaka H. Varotsos P. Sarlis N. Skordas E. (2004) Proc. Jpn. Acad., Ser. B 80, 283–289 (the accompanying paper).

[12] Varotsos P. Sarlis N. Skordas E. (2001) Practica of Athens Academy 76, 294–321.

[13] Varotsos P. Sarlis N. Skordas E. (2002) Acta Geophys. Pol. 50, 337–354.

